# Adsorption of Cellulase on Wrinkled Silica Nanoparticles with Enhanced Inter-Wrinkle Distance

**DOI:** 10.3390/nano10091799

**Published:** 2020-09-10

**Authors:** Aniello Costantini, Virginia Venezia, Giulio Pota, Aurelio Bifulco, Valeria Califano, Filomena Sannino

**Affiliations:** 1 Department of Chemical Engineering, Materials and Industrial Production, Università degli Studi di Napoli Federico II, P.le Tecchio 80, 80125 Napoli, Italy; anicosta@unina.it (A.C.); virginia.venezia@unina.it (V.V.); giulio.pota@unina.it (G.P.); aurelio.bifulco@unina.it (A.B.); 2 Istituto Motori, Italian National Research Council, via G. Marconi 4, 80125 Napoli, Italy; 3 Department of Agricultural Sciences, Università degli Studi di Napoli Federico II, Via Università 100, 80055 Portici, Italy; fsannino@unina.it

**Keywords:** enzyme immobilization, cellulase, wrinkled silica, mesoporous nanoparticles

## Abstract

Mesoporous silica materials offer a unique opportunity for enzyme immobilization thanks to their properties, such as tuneable pore size, large surface area and easy functionalization. However, a significant enhancement of cellulase enzyme activity entrapped inside the silica pores still represents a challenge. In this work, we immobilized cellulase by adsorption on wrinkled silica nanoparticles (WSNs), obtaining an active and stable biocatalyst. We used pentanol as co-solvent to synthesize WSNs with enhanced inter-wrinkle distance in order to improve cellulase hosting. The physical-chemical and morphological characterization of WSNs and cellulase/WSNs was performed by thermogravimetric (TG), Fourier transform infrared (FT-IR), and transmission electron microscopy (TEM) analyses. The obtained results showed that this matrix generates a favourable microenvironment for hosting cellulase. The results of the catalytic assays and operational stability confirmed the key role of size, morphology and distribution of the pores in the successful outcome of the cellulase immobilization process. The immobilization procedure used allowed preserving most of the secondary structure of the enzyme and, consequently, its catalytic activity. Moreover, the same value of glucose yield was observed for five consecutive runs, showing a high operational stability of the biocatalyst.

## 1. Introduction

Over the last Century, the scientific community have devolved a huge interest to address the problems of fossil fuel depletion and climate change [1]. These issues make the alternative sustainable and renewable energy sources research necessary [2]. In this frame, bioethanol and biodiesel represent a very good alternative to fossil fuels. Cellulose represents a broad platform to obtain chemicals and energy. Cellulose is a polymer composed of glucose units linked by 1,4-β-glycosidic bond [3]. It is present in large amount in the biosphere [4,5] and is the main component of lignocellulosic biomass. The use agricultural and forestry waste as raw materials is a valid strategy to produce bioethanol.

Enzymatic hydrolysis of cellulose is very advantageous in respect to chemical hydrolysis, due to the milder operative conditions. Cellulase are a class of enzymes devoted to cellulose hydrolysis. They are composed by three different classes of enzymes, endoglucanase, exoglucanase and β-glucosidase, which act synergistically and sequentially on the polymeric chains of cellulose. The use of enzymes has some drawbacks, due to their high cost and their fragile nature. To address these problems, one possible solution is their immobilization on insoluble supports.

Mesoporous silica can be used for many applications such as drug delivery, fluorescence biological probes [6,7] and are suitable supports for enzyme immobilization [8,9,10]. They have high chemical, mechanical and thermal stability. Their high surface area and pore volume allow for large enzyme loadings and a good dispersion inside the pores. In this regard, the matching between the pore size and the molecular diameter of the enzyme plays a key-role: the pores must be large enough to host the enzyme and maintain its mobility/flexibility within the cavities. Still there should be space enough to allow the substrate diffusion of to the active sites of the enzymes. However, too large pores can give rise to enzyme leaching and the loss of its conformational flexibility due to crowding effects. Takimoto et al. [11] immobilized cellulase from *Trichoderma viride* in Santa Barbara Amorphous 15 (SBA-15) with different pore size—5.4, 8.9 and 11 nm. They demonstrated that the activity of immobilised cellulase were maximized by the matching of the silica pore size and the molecular size of cellulase. In fact, when the pore size was just enough to host the cellulase, the enzyme remained close to the pore entrance, allowing an easy accessibly of its active site. Chen et al. [12] synthesized two mesoporous silica with different pore size of 17.6 nm (MS-17.6) and 3.8 nm (MS-3.8). They used these mesoporous materials to immobilize cellulase from *Acremonium* by pure physical adsorption. MS-3.8 displayed a higher specific activity than MS-17.6. Again, this was attributed to the sticking of the enzyme at the pore entrance, increasing the accessibility of the active site and preserving the protein native structure. In MS-17.6, the dense and ordered arrangement inside the pores hampered the conformational flexibility of cellulase, lowering its activity.

Surface functionalization is often necessary to improve the interactions between the enzyme and the silica support, and to increase the loading, since cellulase has large hydrophobic areas. Many researchers have functionalised mesoporous silica with organic compounds such-as vinyl (CH=CH_2_) [13] and thiol (–SH) in SBA-15 and FDU-12 (FDU coming from Fudan University) [14,15]. FDU-12 materials, due to their big pores and three-dimensional connectivity, represent a valid support to host bulky enzymes such as cellulase, avoiding mass diffusion limitation. Vinyl functionalized FDU-12 showed significant improvements of the cellulase activity [16]. The hydrophobic characteristics within silica materials induced a high loading amount and created a favourable microenvironment for catalysis. Cellulase immobilized on functionalized mesoporous cellular foam (MCF) showed higher activity than the free one due to the proven activity of the functional groups on surface modified MCF toward the hydrolysis of carboxymethylcellulose sodium salt (CMC) [17]. Although there are many studies on the immobilization of cellulase into mesoporous silica [18], significant enhancement of immobilized cellulase activity remains a challenge. Actually, adsorption from a mixture is difficult to control due to the varying kinetics of adsorption, variations in the degree of unfolding, and competitive binding effects [19]. Furthermore, cellulase establishes a strong interaction with silica. This interaction inhibits cellulose hydrolysis [20].

The successful immobilization of enzymes on porous silica samples is largely determined by the physicochemical properties of the porous hosts, such as morphology, particle size, composition and pore size [21]. For example, Hisamatsu et al. [22] studied the adsorption of α-amylase on mesoporous silica with different pore sizes, morphology and surface properties. They found that the amount of encapsulated enzyme increased with increasing pore size of the silica support. The presence of large pores with disordered arrangements and smaller particles size favoured the enzyme adsorption. Lei et al. [23] studied the influence of SBA-15 support morphology on the immobilization behaviour of lysozyme finding that rod-like SBA-15 exhibited higher loadings and a faster adsorption rate than spherical SBA-15. Zhou et al. [24] investigated the influence of particle morphology on the immobilization of Candida rugosa lipase (CRL). CRL immobilization on vesicle-like silica possessed higher thermal stability and reusability compared to CRL immobilized on rod-like silica.

Recently, silica nanoparticles with radial wrinkle structure (WSNs) were synthesized [25,26]. WSNs have wrinkles that widens radially outward enhancing the accessibility of functional materials inside the pores and reducing pore blocking. They were used for lipase [27] and β-glucosidase [28,29,30] immobilization. Lipase immobilized in WSNs showed higher activity than the free enzyme. The better performance was attributed to the radially aligned mesopores of WSNs, allowing dispersion of dispersing active catalytic sites on large internal surface and pores [27]. The immobilised β-glucosidase showed better catalytic performance than free enzyme and high thermal and operational stability. This was attributed to the peculiar morphology of the pores and their hierarchical structure allowing for optimal hosting of β-glucosidase and avoiding diffusion problems of the substrate [28,29].

WSNs are synthetized by the microemulsion method. The microemulsion generally contains iso-propanol as co-solvent. It was found that the chain length of the co-solvent influences the inter-wrinkle distances [25]. In particular, by increasing the chain length of the co-solvent, the inter-wrinkle distance increases. In our study, pentanol was used as co-solvent to synthesize WSNs with enhanced inter-wrinkle distance. The aim was to increase the loading and activity of cellulase on mesoporous silica nanoparticles without the need to functionalize the support with organic compounds.

## 2. Materials and Methods

### 2.1. Materials

Tetraethylorthosilicate (TEOS), urea, cetyltrimethylammonium bromide (CTAB), cyclohexane, pentanol, acetone, 37% hydrochloric acid solution, citric acid, and trisodium citrate were used to synthesize WSNs with pentanol (WSN-p). WSNs with isopropanol (WSN-ipa) were prepared in the same way as WSN-p, but using isopropanol instead of pentanol. All chemicals were purchased from Sigma-Aldrich (Milan, Italy). For enzyme physical adsorption and kinetic measurements, cellulase from Tricoderma Reesei and glucose oxidase-peroxidase (GOD-POD) assay kit from Sigma-Aldrich (Milan, Italy) were used. Citrate buffer solution at pH 5.0 was prepared by dissolving 3.9 mg of citric acid in 205 mL of bidistilled water, and 8.7 mg of trisodium citrate in 295 mL of bidistilled water. The two solutions were mixed and the volume was brought to 1 L by diluting with bidistilled water.

### 2.2. Nanoparticle Synthesis

WSNs synthesis route was carried out following the procedure reported in literature [25], using a water/surfactant/oil ternary system as structural template. CTAB, aqueous urea and pentanol or isopropanol/cyclohexane were used as surfactant, water phase, co-solvent and oil phase, respectively. TEOS was chosen as silica precursor. The reaction mixture was vigorously stirred for 30 min at room temperature, heated up to 70 °C and kept overnight in a closed vessel to avoid solvent evaporation. Three centrifugations were carried out to recover the nanoparticles. The surfactant was removed by chemical extraction with ethanol/hydrochloric acid mixture. A certain amount of WSN-ipa was calcined (WSN-Calc) into a tubular oven (2 °C/min from 25 to 500 °C and isotherm at 500 °C for 4 h) (Memmert, Buechenbach, Germany) to remove the surfactant that was not eliminated by chemical extraction.

### 2.3. Chemical and Physical Characterization

The morphology of bare WSN and biocatalyst (cellulase/WSN) was evaluated by transmission electron microscopy (TEM) (PHILIPS EM208S microscope equipped with a Mega View camera for digital acquisition of images (FEI, Hillsboro, OR, USA).

The organic content of each sample was estimated by thermogravimetric analysis (TGA) (TA Instrument, New Castle, DE, USA) using a thermogravimetric apparatus Q600SDT, under air atmosphere, in a temperature range between 25 and 800 °C and at heating rate of 10 °C/min. Samples mass were 10 mg, put in a platinum crucible.

A Nicolet Instrument Nexus model equipped with a DTGS KBr (deuterated triglycinesulfate with potassium bromide windows) detector (Thermo Scientific, Waltham, MA, USA) was used to perform the FT-IR investigation. IR spectra were recorded in the 4000–400 cm^−1^ range with a 2 cm^−1^ resolution. The IR spectrum of each sample was corrected for the spectrum of blank KBr by mixing 199 mg of KBr and 1 mg of dried samples powders and pressing into pellets of 13 mm diameter.

### 2.4. Cellulase Immobilization

The adsorption procedure followed the route described by our research group in a previous work [29]. Herein, physical immobilization of cellulase into WSN-p was carried out changing adsorption time, enzyme/support ratio and temperature. In the first case, each sample was prepared by dissolving 10 mg of enzyme in 8 mL of a 20 mM citric acid/sodium citrate buffer solution at pH 5.0. Then, in order to obtain enzyme-to-support weight ratio of 0.5, 2 mL of buffer containing 20 mg of suspended WSN-p were added to the enzyme solution. The final colloidal solutions were gently stirred at 25 °C for 1 h, 3 h, 6 h and 24 h, respectively. In the second case, physical adsorption was carried out in the same conditions for 24 h but we fixed four different enzyme-to-support weight ratios: 0.25, 0.50, 0.75, 1. In the third case, the adsorption temperature was set to 25, 30, 40 and 50 °C using an enzyme-to-support weight ratio of 0.5.

The resulting biocatalysts were recovered by centrifugation at 12,500 rpm for 15 min and washed twice with the buffer to eliminate the non-adsorbed enzyme molecules.

Further cellulase was adsorbed into WSN-ipa (30 °C, enzyme-to-support weight ratio 0.50, 24 h), following the same procedure described above. Moreover, the enzyme was immobilized into WSN-Calc (enzyme-to-support weight ratio 0.50, 24 h).

The TGA curves of all the samples, not reported in this paper, showed a first weight loss below 200 °C related to water desorption and a second weight loss starting from 200 °C. The latter can be attributed to different causes, such as the condensation of surface Si–OH to form Si–O–Si bonds for WSNs, the degradation of organic component, i.e., the progressive deamination, decarboxylation, and depolymerisation arising from the breaking of polypeptide bonds for biocatalyst [29]. We evaluated the amount of immobilized enzyme (IE), subtracting the weight loss of each biocatalyst to the one of bare support in the range between 200 and 800 °C. Then, we determined the immobilization yield of cellulase (*YI*) according to the following equation:

(1) $$YI=\frac{E}{E_{0}}$$

where *E*_0_ is the weight of the enzyme used in the adsorption step and *E* that of the adsorbed enzyme.

### 2.5. Catalytic Assays

The kinetic behaviour of immobilized cellulase was assessed by measuring the concentration of glucose produced by the hydrolysis of carboxymethylcellulose sodium salt (CMC), used as substrate. The specific activity is defined as μmoles of produced glucose over time (minutes) per gram of immobilized enzyme. CMC was dissolved gradually in 20 mM citric acid/sodium citrate buffer (at 60 °C, under vigorous stirring) to have a final concentration of 20 mg/mL. Then, a specific volume of this solution was added to an equal volume of a 20 mg/mL cellulase/WSN-p buffer suspension, in order to reach a final enzyme and substrate concentration of 1 mg/mL and 10 mg/mL, respectively. The reaction was carried out at 50 °C, under gentle stirring. Precise volumes of the reaction mixture were withdrawn at fixed time (30 min, 1 h, 2 h, 6 h and 24 h) put in oven (100 °C, 10 min) to thermally inactivate the biocatalyst and finally centrifuged (11,500 rpm, 10 min). The supernatant was recovered and its glucose concentration was determined. A similar procedure was set up for testing the activity of free cellulase. A specific volume of 20 mg/mL CMC solution was mixed to an equal volume of a 2 mg/mL cellulase buffer solution to obtain the reaction mixture. Just the thermal inactivation of the biocatalyst was needed for all the withdrawn volumes to be ready for the analysis.

Glucose concentration was evaluated by glucose (GO) assay kit, following the D-glucose oxidase–peroxidase method [31]. The quantity of glucose originating from the reaction was estimated by adding 2 mL of glucose-measuring reagent to 1 mL of quenched reaction mixture, previously diluted. The resulting mixture was kept at 37 °C for 30 min in a thermostatically controlled water bath. Absorbance (OD) at 540 nm was measured by a spectrometer instrument (Perkin Elmer Instruments, Lambda 25 UV/Vis, PerkinElmer, Inc., Waltham, MA, USA). Each experiment was performed in triplicate.

### 2.6. Operational Stability

The immobilized sample cellulase/WSN-p was analysed in consecutive 24 h reaction cycles to evaluate the biocatalyst reusability. Each reaction was carried out under standard assay conditions (50 °C, pH 5 and [CMC] 10 mg/mL). After each cycle, the solution was centrifuged and the supernatant was analysed by UV-Vis analysis to determine glucose concentration. The hydrolysis yield after the first cycle of the reaction was used as the reference (100% conversion).

## 3. Results and Discussion

### 3.1. Choice of Supports

**Figure 1:**
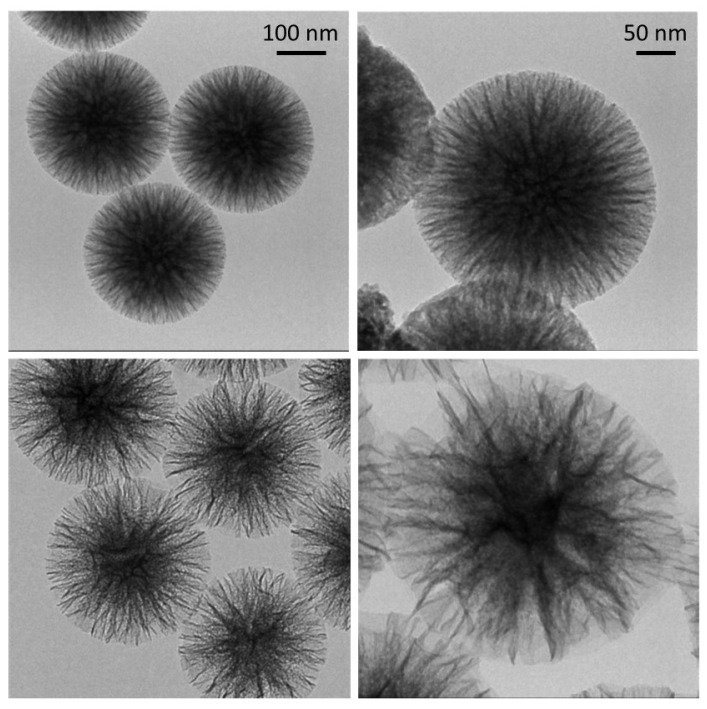
Transmission electron microscopy (TEM) micrographs of WSNs with isopropanol (WSN-ipa) (top) and WSNs with pentanol (WSN-p) (bottom) at lower (left) and higher (right) magnification.

The morphology of the two synthesized siliceous supports is illustrated in the TEM micrographs of Figure 1.

In both cases, the particles are made of silica fibres that spread radially inside out of the particle. However, the WSN-p show a lower density and appear more branched. The inter-wrinkle distance is greater.

**Figure 2:**
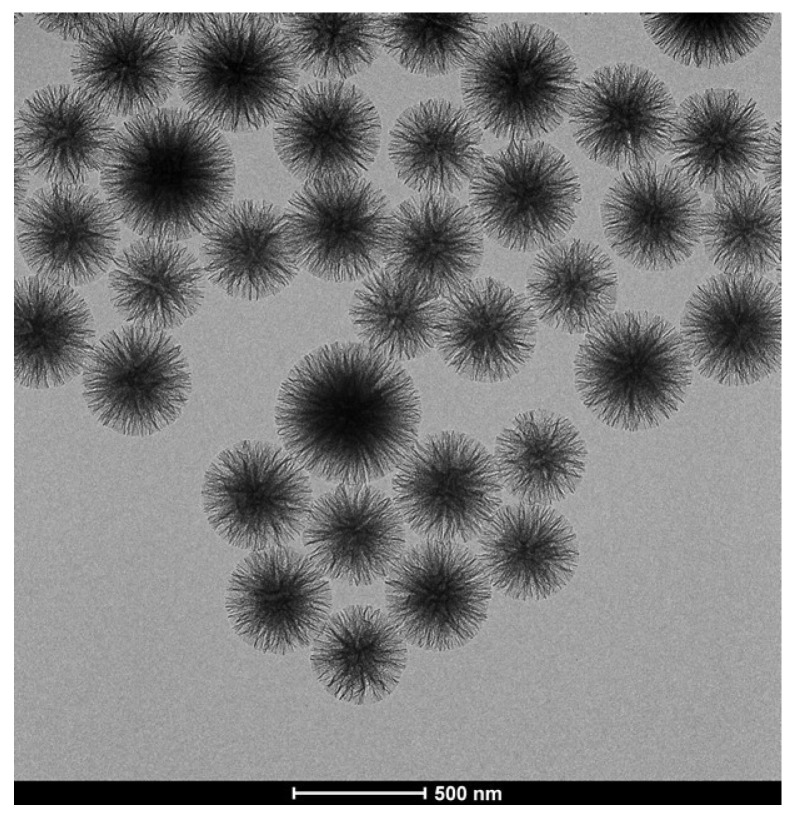
TEM micrographs of WSN-p at low magnification.

Figure 2 shows the TEM micrograph of WSN-p at lower magnification. It can be observed that the particles are quite monodisperse, with sizes ranging from about 300 to about 500 nm.

**Figure 3:**
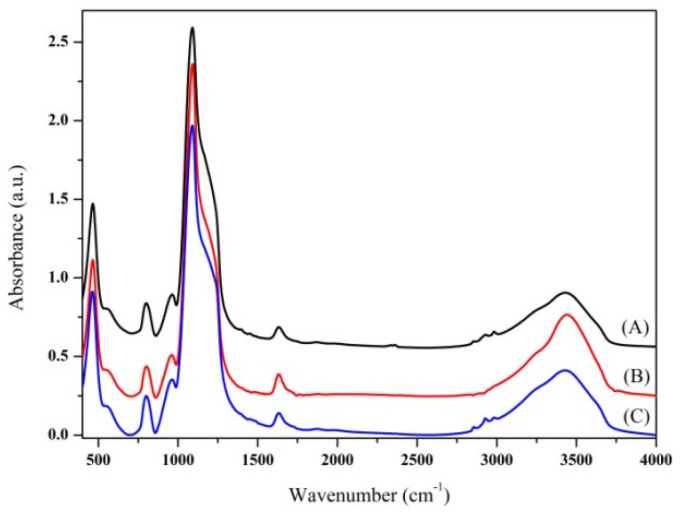
Fourier transform infrared (FT-IR) spectra of (A) WSN-p, calcined WSN (WSN-calc) (B) and WSN-ipa (C).

WSN-ipa were also calcined, with the aim of removing the surfactant not extracted by the acid treatment and allow for more space between the silica fibres. From the spectra shown in Figure 3, no particular difference is noted in the silica structure of the three samples. The calcined sample shows the disappearance of the bands related to organic material, at about 1400 cm^−1^ and between 2800 and 3000 cm^−1^.

**Figure 4:**
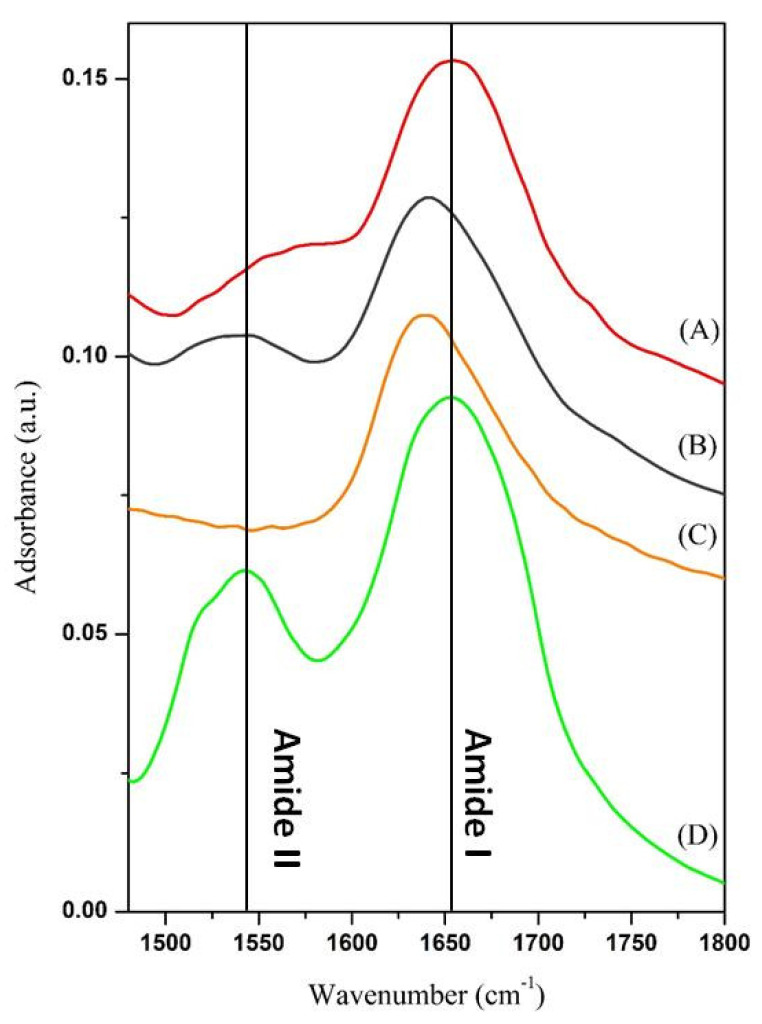
FT-IR spectra of cellulase adsorbed on WSN-p (A), WSN-calc (B) and WSN-ipa (C) and free cellulase (D).

To choose the best support, preliminary adsorption experiments were performed at 25 °C and the enzyme/support complex examined by FT-IR in the region 1480–1800 cm^−1^. In fact, two of the characteristic bands of the polypeptide structure fall in this range, the amide I and the amide II [32]. These bands, in particular the amide I, have positions that are very sensitive to the secondary structure of the enzyme. In fact, the amide I band originates from the stretching of the carbonyl groups of the peptide bond, and is given by the overlap of a set of vibration modes. Each of these modes of vibration is linked to a particular element of secondary structure of the enzyme: α-helices, β-sheets, turns, disordered structures and intermolecular aggregates. During adsorption, proteins can undergo conformational changes due to interactions with the matrix surface or protein-protein interactions. The presence of intermolecular aggregates, with vibration modes at lower wave numbers (about 1610 cm^−1^) is an indication of the incipient denaturation of the protein native structure and is therefore deleterious for its catalytic activity [33]. The FT-IR spectra of the immobilized and free cellulase are displayed in Figure 4.

The spectra of cellulase immobilized on WSN-ipa, either calcined or not, show a displacement of the amide I band toward lower wavenumber respect to free cellulase. This is an index of aggregation/denaturation of enzyme molecules, to the detriment of their catalytic activity. Calcination does not improve this situation. By contrast, for cellulase immobilized on WSN-p amide I band is about in the same position as free cellulase. This phenomenon can be related to the different morphology of the particles. In fact, from the chemical point of view, there are no differences between the two types of nanoparticles (see Figure 3), while morphologically they are significantly different. The greater distance between the fibres is likely to favour the adsorption of unmodified cellulase molecules. Based on these preliminary results, WSN-p were chosen as support for subsequent studies.

### 3.2. Optimization of the Adsorption Conditions

First, the adsorption time was optimized by conducting the process at various times ranging from 1 h to 24 h at 25 °C. In this case, the concentrations of enzyme and nanoparticles used was 1 mg/mL and 2 mg/mL respectively. The adsorption equilibrium was reached at 24 h. At this time, the amount of adsorbed enzyme was 70 mg per gram of support. The adsorption time was therefore set at 24 h.

**Figure 5:**
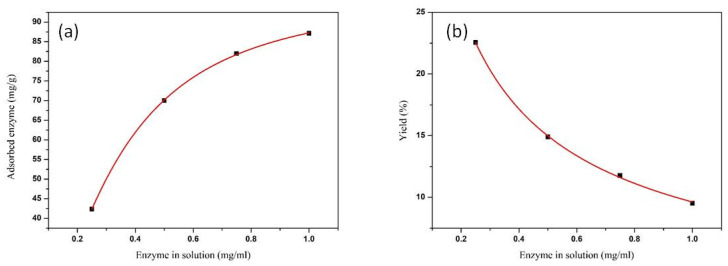
Adsorption isotherm (squares) and Langmuir fit (line) (**a**), and adsorption yield vs. the initial concentration of the enzyme in solution (squares) fitted with an exponential decay function (line) (**b**).

To favour the economy of the overall process of enzymatic catalysis at an industrial level, it is important to minimize the enzyme waste. The adsorption should take place with the minimum possible amount of enzyme that assures high load and a high yield of immobilization. For this purpose, adsorption tests were carried out varying the enzyme concentration from 1 mg/mL to 0.25 mg/mL. The results are shown in Figure 5. Figure 5a shows the adsorption isotherm fitted with a Langmuir function. Langmuir adsorption isotherm model assumes monolayer adsorption. Monolayer adsorption, as the most probable mechanism involved in the adsorption of cellulase on amorphous silica, has already been observed [23]. Figure 5b represents the adsorption yield vs. the initial concentration of the enzyme in solution.

The adsorbed amount increased with cellulase concentration up to 1 mg/mL, and the onset of the adsorption plateau amounted to 90 mg/g of support (at cellulase concentration of 1 mg/mL). The immobilization yield decreased with increasing enzyme concentration. A concentration of 0.50 mg/mL was chosen for subsequent experiments because it makes a good compromise between the immobilization yield and the amount of immobilized enzyme.

**Figure 6:**
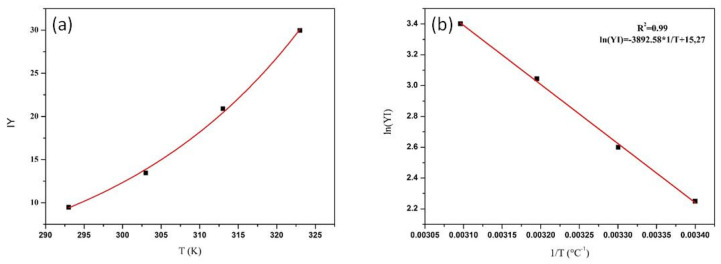
Yield of immobilization plotted versus T (squares) and exponential growth fit (line) (**a**) and Arrhenius plot (**b**).

The effect of temperature on the adsorption behaviour of cellulase (bulk concentration 0.50 mg/mL, adsorption time 24 h) is shown in Figure 6a.

The adsorbed amount increased from 25 to 50 °C according to an Arrhenius-like trend. This indicates that the adsorption of cellulase on WSNs in endothermic in nature [12,34]. Adsorption at higher temperatures has not been analysed because enzymes are normally prone to thermal deactivation by denaturation. In order to estimate the activation energy of adsorption, the data were treated according to an Arrhenius plot, as shown in Figure 6b. From the linear fit, the adsorption energy was found to be 25 kJ/mol, which is comparable to the energy involved in hydrogen bonding (10–40 kJ/mol). Hence, the driving force of cellulase adsorption into WSN is probably hydrogen bonding between cellulase polar residues and silanol groups on the silica surface [20]. This is important, as adsorption from a mixture of enzymes is difficult to control. In fact, cellulase is composed of at least three enzymes with different chemical-physical properties. In particular, they have different shapes, isoelectric points and hydrophobic characteristics. If the driving force for adsorption were electrostatic interaction, there would probably be preferential adsorption effects due to the different isoelectric points of the cellulolytic enzymes. Similarly, the hydrophobic interaction would give rise to preferential adsorption due to the different shape and hydrophobic characteristics of the enzymes. The hydrogen bond instead ensures the adsorption of a uniform layer from a cellulase enzyme mixture, being all cellulolytic enzymes rich in hydrogen bonding groups.

**Figure 7:**
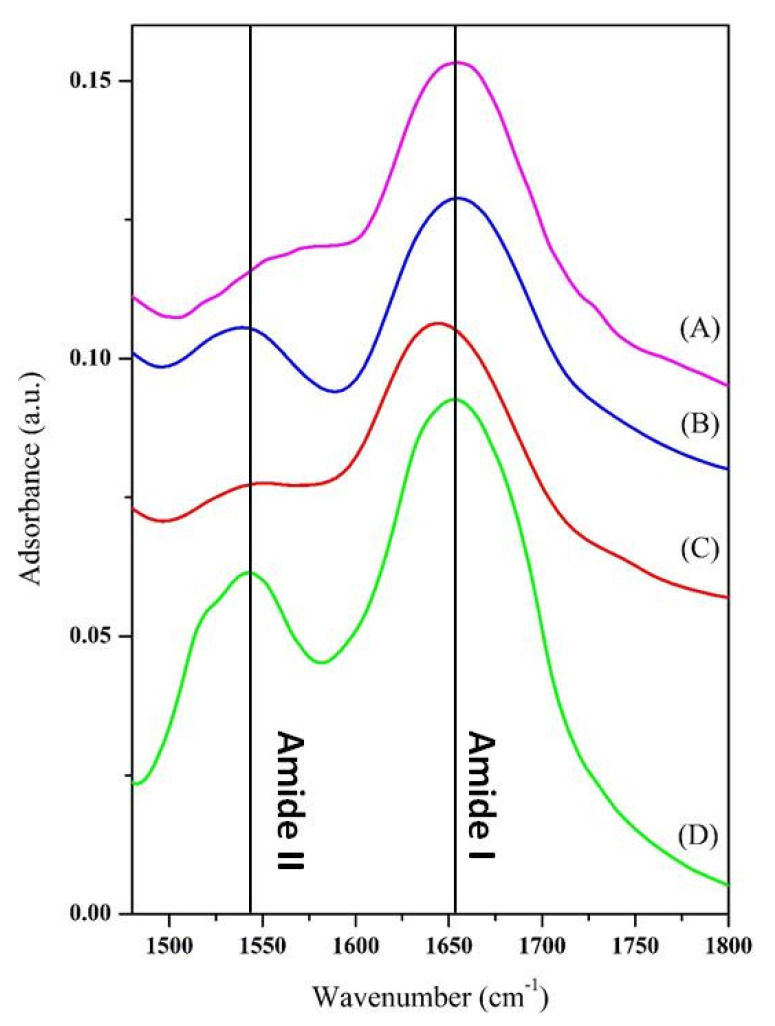
FT-IR spectra of cellulase adsorbed in WSN-p at 30 (A), 40 (B) and 50 °C (C), and of free cellulase (D).

The data indicate that the highest adsorption in the temperature range examined occurs at 50 °C. However, increased adsorption does not always coincide with increased enzyme activity, which can be denatured by thermal or crowding effects. For this reason, the FT-IR spectra of the three samples were recorded, and are shown in Figure 7 in the region 1480–1800 cm^−1^.

The spectrum of cellulase adsorbed at 50 °C shows an offset of the amide band I towards lower wave numbers compared to free cellulase, indicative of denaturation/aggregation of the molecules by means of intermolecular hydrogen bonds. The amide band II is also modified. The other spectra appear instead very similar to those of free cellulase. In particular, the spectrum of cellulase adsorbed at 40 °C shows a similarity with that of free cellulase in the position and relative intensity of the amide bands I and II. The temperature chosen to carry out the adsorption was then 40 °C, at which the native structure of the polypeptide shows little or no modification and the loading (100 mg/g of support) is higher than at 25 and 30 °C.

### 3.3. Catalytic Assays

**Figure 8:**
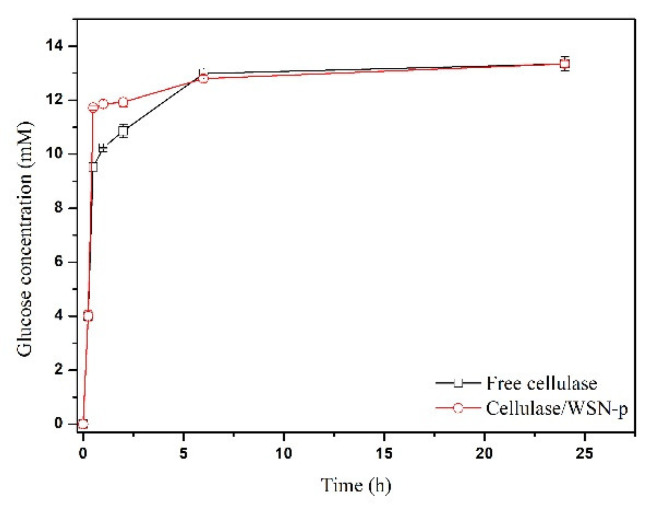
Glucose concentration versus time for free cellulase (square) and cellulase/WSN-p (circle). Data are reported with error bars.

The kinetics of CMC hydrolysis catalysed by the free enzyme and the biocatalyst cellulase/WSN-p was evaluated. Figure 8 shows the time course of the reaction, reporting glucose concentration (mM) vs. time (h).

**Figure 9:**
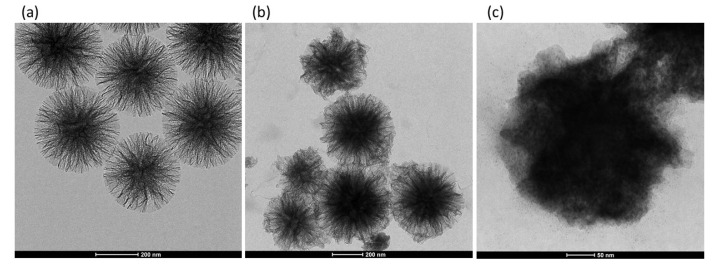
TEM micrographs of WSN-p (**a**) and BG adsorbed on WSN-p (BG/WSN-p) (**b**,**c**).

The activity of the free enzyme evaluated at 15 min is 267 µmol/g∙min. The immobilized enzyme retained 100% of the free enzyme activity. Achieving total retention of cellulase activity is not trivial. For example, Zhang et al. [35] covalently immobilized cellulase on silica gel substrate, retaining only 7% of its specific activity in the hydrolysis of CMC. Chen et al. [12] immobilized cellulase on mesoporous silica with different pore size, obtaining at the best a retention of 63.3% of free cellulase activity. Takimoto et al. [11] immobilized cellulase in SBA-15 with different pore size: the best biocatalyst preserved 67.5% of the activity of the free form. In two cases, the activity retention achieved was more than 90%, for cellulase immobilized by sol-gel encapsulation in mesoporous silica functionalized with methyl groups, which give a certain hydrophobicity [36] and for cellulase immobilized on commercial fumed silica, nonporous in nature [37]. Finally, in two cases the activity of immobilized cellulase was found to be higher than that of the free one [2,17]. In both cases, cellulase was immobilized by covalent bonding to mesoporous silica through amino functionalization of the silica surface followed by crosslinking with glutaraldehyde. The reason for this increase in activity was due to a certain activity of functional groups on the silica surface toward the hydrolysis of CMC [17]. In our case, the total preservation of the enzymatic activity indicates that physical immobilization did not change the native conformation of the protein, as already indicated by FT-IR analysis, and that there are no diffusion limitations of the substrate to the active site of the enzyme. This is due to the particular morphology of WSNs, with radial pore channel size increasing from the interior to the surface. This hinders pore blocking, allows the enzyme to settle in the interior of the pores where the interactions with the walls are maximized, and still there is room for the substrate to easily diffuse. To endorse this hypothesis, Figure 9 shows the TEM micrograph of WSN before and after cellulase adsorption. For BG adsorbed on WSN-p (BG/WSN-p), it is possible to observe two populations of nanoparticles. One shows an increase in electron density in the inner of the pores, due to the presence of the adsorbed enzyme, whereas the periphery of the nanoparticles appears quite unchanged (Figure 9b). Most BG/WSN-p exhibit this aspect. However, there is also a population of BG/WSN-p that appear fuller and in which it is possible to observe the presence of the enzyme also on the external surface Figure 9c).

**Figure 10:**
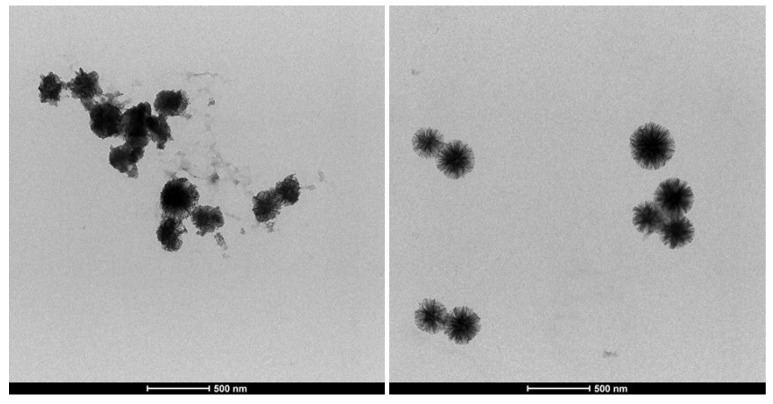
TEM micrographs of BG/WSN-p at low magnification.

The two kinds of nanoparticles are also shown in TEM images at low magnification of Figure 10.

The hydrolysis catalysed by immobilized cellulase exhibits an initial burst: at 30 min, the glucose concentration reaches more than the 90% of the final value. However, the reaction promoted by the immobilized enzyme approaches more slowly the equilibrium value, if compared to the behaviour of free cellulase. This evidence can be attributed to product inhibition caused by the accumulation of the glucose inside the pores of the support [29]. The curves in Figure 8 share the same asymptotic value (about 13 mM of glucose concentration), corresponding to CMC percentage conversion of 56%.

### 3.4. Reusability Tests

**Figure 11:**
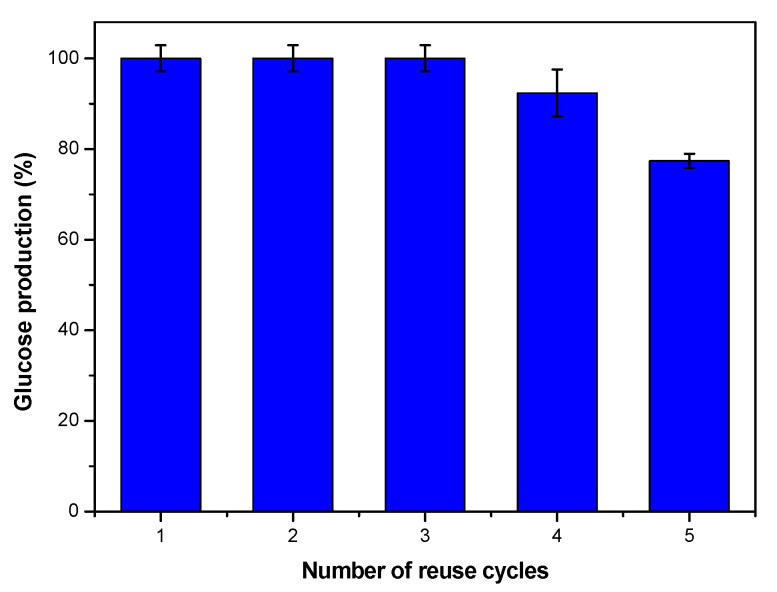
Histograms showing the glucose production (%) over the number of reuse cycles of cellulase/WSN-biocatalyst (data are reported with error bars).

Reusability of cellulase immobilized by physical adsorption into mesoporous silica materials was not generally tested. The reusability of an enzyme catalyst is a very important factor, which contributes to reducing overall costs by offsetting the high production cost of the enzyme. Figure 11 shows the results of reusing the biocatalyst in 5 consecutive 24 h cycles. The amount of glucose that has formed in each cycle is expressed as a percentage of the maximum obtained from the catalytic assay. Cellulase/WSN-p exhibits a good operational stability: glucose production is about 100% up to the third reuse, while in the fourth one, it shows a loss of about 8% of conversion, and in the fifth one, the loss drops of about 20%, which is still enough for reuse. Similar results were observed for cellulase immobilized by physical adsorption on Si wafers [20], Poly (vinyl alcohol-co-ethylene) nanofibrous membranes [38] and on commercial activated carbon [39].

The loss of catalytic performance during reuse can be caused by enzyme leaching/desorption, denaturation during repeated use or physical loss of the biocatalyst during separation and washing processes after each cycle.

## 4. Conclusions

Cellulase was immobilized by adsorption on wrinkled silica nanoparticles with enhanced inter-wrinkle distance. The physical-chemical characterization of the biocatalyst demonstrated the improvement induced by the increase of the inter-wrinkle distance on hosting cellulase. The adsorption energy was found to be 25 kJ/mol, pointing out that the driving force of cellulase adsorption into WSN is probably hydrogen bonding between cellulase polar residues and silanol groups on the silica surface. The activity of cellulase/WSNs was determined in the hydrolysis of carboxymethyl cellulose. The immobilized cellulase had the same activity as the free enzyme and showed a good operational stability. Therefore, this study represents, both for the simplicity of the biocatalyst preparation and its good performance and reusability, a step toward the large-scale hydrolysis of cellulose for biofuel production.
